# The role of an advanced practice midwife in perinatal mental health: Outlining the process of role development and implementation

**DOI:** 10.18332/ejm/189954

**Published:** 2024-07-05

**Authors:** Lena Sutter, Felicitas Rewicki, Daniel Surbek, Sebastian Walther, Régine Goemaes, Lynn Alexia Huber, Eva Cignacco

**Affiliations:** 1 Department of Obstetrics and Gynecology University Hospital of Bern, Bern, Switzerland; 2 University of Applied Sciences, School of Health Professions, Bern, Switzerland; 3 Hirslanden Klinik Linde, Biel, Switzerland; 4 University Hospital of Psychiatry and Psychotherapy, Bern, Switzerland; 5 Department of Public Health and Primary Care, Academic Centre for Nursing and Midwifery, Leuven, Belgium

**Keywords:** perinatal mental health, advanced practice midwife, PEPPA framework, role development and implementation, perinatal mental health disorders

## Abstract

**INTRODUCTION:**

Perinatal mental health disorders (PMDs) are a global health concern. In industrialized countries, the prevalence of PMDs is estimated to be 20%, and they are associated with serious negative effects for women, their children and their families, along with high societal costs related to long-term impacts. In Switzerland, the PMD detection rate during obstetrical healthcare provision is very low (1–3%), and specialized healthcare services are limited. This study aimed to develop and implement an advanced practice midwife (APM) role at a Swiss obstetrics and gynecology hospital using the PEPPA framework to provide adequate screening and first-consultation services.

**METHODS:**

The study uses a qualitative approach and follows the research stages using the 8-step from the participatory, evidence-based, patient-focused process for advanced practice nursing role development, implementation and evaluation (PEPPA) framework to develop and implement the APM role.

**RESULTS:**

Utilizing the PEPPA framework, we were able to develop, implement, and evaluate the APM role in the field of perinatal mental health. Through appropriate screening and first-consultation services, we were able to identify affected women early and facilitate treatment.

**CONCLUSIONS:**

In addition to stakeholder engagement and interprofessional collaboration, PEPPA serves as a beneficial framework for the process of role development, implementation, and evaluation in the midwifery profession. This study aims to assist midwives with Master's degrees in establishing corresponding roles within their practice areas, thereby enhancing care delivery. Furthermore, the current APM approach is intended to be continuously evaluated to gain new insights into its effectiveness.

## INTRODUCTION

Perinatal mental health disorders (PMDs) are a major global health concern^1^. In industrialized countries, the prevalence of women with mental health disorders during the perinatal period is estimated to be 20%^1,2^. In Switzerland, around 17% of women are affected by PMDs^3^. If not appropriately treated, PMDs can have severe adverse effects on women and their children^2^. The consequences of PMDs include an increased risk of preterm birth, lower infant birth weight, higher cesarean section rates, and increased maternal substance use^4^. PMDs can also negatively influence the mother–child relationship, which may increase the risk of developmental disorders in children^5^ and family relationship dysfunctions^6^.

Furthermore, suicide is the leading cause of maternal death in the first year after birth^7^. In the general population, more than 50% of individuals with mental disorders remain untreated^8^. It can be estimated that an equal or larger number of women may be affected by PMDs due to specific barriers that prevent women from accessing perinatal mental health (PMH) services, such as stigma, a lack of systematic PMD screening, and structural barriers within the healthcare system^9^.

Switzerland lags behind Western peers in screening and treating PMDs, with a national detection rate of 1–3% and lacking published guidelines for PMD care^3^. Compared to other countries, PMH care in Switzerland lacks focus and thus is not sufficiently specialized to properly care for women during this perinatal period^3^.

Several studies have indicated that midwives with adequate education are capable of delivering appropriate care to women with PMDs and, if necessary, referring them to mental health providers for further interventions^10^. In the United Kingdom, and in Ireland, midwives work in advanced practice midwife (APM) roles as specialist maternal mental health midwives^11^.

Several studies have demonstrated the clinical benefits of advanced practice nurses (APNs). Evidence shows that APN use leads to improved patient outcomes, reduced hospital admissions and readmission rates, decreased morbidity, and improved patient satisfaction and quality of life^12^.

Unlike developments in the nursing profession, few countries have implemented APM roles. Nevertheless, the benefits of APM services have also been confirmed^11^.

In Switzerland, midwives had, for the first time in 2017, the opportunity to pursue a Master of Science degree following their Bachelor of Science, enabling them to assume Advanced Practice Midwifery (APM) roles. However, the roles of APMs in Switzerland lack legal regulation under the current federal health profession legislation^13^.

The Swiss Professional Conference of Midwifery defines an APM as an accredited practicing midwife with a Master of Science degree, in-depth expertise in a specific practice domain, research skills, and advanced leadership competences. APMs provide continuous woman- and family-centered care in complex clinical situations with high autonomy, efficiency, and accountability. They work in various settings, promoting and coordinating interprofessional collaboration within health and social systems. Moreover, APMs contribute to producing scientific knowledge and communicating it to diverse audiences. They also conceptualize and implement accessible, equitable, cost-effective, innovative solutions supporting health promotion and prevention. Overall, APMs improve the quality of care, contribute to public health, and advance midwifery as an academic profession^13^.

The APM role described in this article was developed as part of a Master’s thesis using the participatory, evidence-based, patient-centered process to develop, implement, and evaluate the APN role (PEPPA). The PEPPA framework was used because it has been successfully utilized nationally and internationally in developing, implementing, and evaluating roles for advanced practice nurses^14,15^. The role was implemented in 2020 at a University Hospital for Obstetrics and Gynecology in cooperation with a University Hospital of Psychiatry and Psychotherapy. The aim of developing and implementing an APM role within PMD care was to improve the early detection, diagnosis, and treatment of PMDs, thereby optimizing the care of PMD-affected or psychologically distressed women, and minimizing risks for mothers, children, and families. So far, this is the first and only advanced midwifery role in Switzerland and is undergoing continuous evaluation. This study shows the process of developing an APM role and the results of its implementation.

## METHODS

### Study design

The study uses a qualitative approach and follows the research stages using the 8-step from the PEPPA framework^16^. This framework comprises a total of nine steps: 1) Define the patient population and describe the current model of care; 2) Identify stakeholders and recruit participants; 3) Determine the need for a new model of care; 4) Identify priority problems and goals to improve the model of care; 5) Define the new model of care and the APN role; 6) Plan implementation strategies; 7) Initiate the implementation plan; 8) Evaluate the APN role and new model of care; and 9) Conduct long-term monitoring of the APN role and new model of care^16^.

### Qualitative data, participants and ethics

In PEPPA Step 1, five interviews were randomly selected from a parallel-running project, *Perinatal Mental Health Care in Switzerland: Unravelling the Perspectives of Affected Women and Health Professionals* (MADRE), within the Obstetrics Research Department of the Bern University of Applied Sciences Health (BFH)^17^. The MADRE study received ethics approval from the Cantonal Ethics Committee for Research, Health and Welfare Directorate of the Canton of Bern, Switzerland, on 21 March 2019 (reference number 2018–02345) ^17^. The women interviewed had a psychiatric (ICD-10-F) diagnosis within the categories of mood disorders, behavioral and personality disorders, or psychosis.

The perspectives of healthcare providers (PEPPA Step 3) were also obtained from notes taken during focus group interviews with different healthcare professionals for the MADRE study. The health professionals were interviewed in three focus groups, with six to eight health professionals participating in each interview. The health professionals included psychiatrists, psychologists, gynecologists, a social worker, a member of the child and adult protection authority, a counselor for mothers and fathers, an APN from the neonatal intensive care unit, the chairperson of the Swiss Postnatal Depression Association, outpatient midwives, family care midwife, and a pediatrician. All individual interviews were transcribed using *f4transkript* transcription software^18^ and analyzed using thematic analysis^19^.

**Figure 1:**
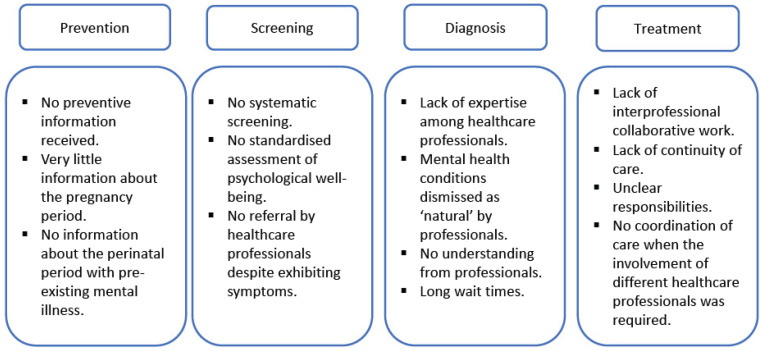
Adapted PMH care pathway

**Figure 2:**
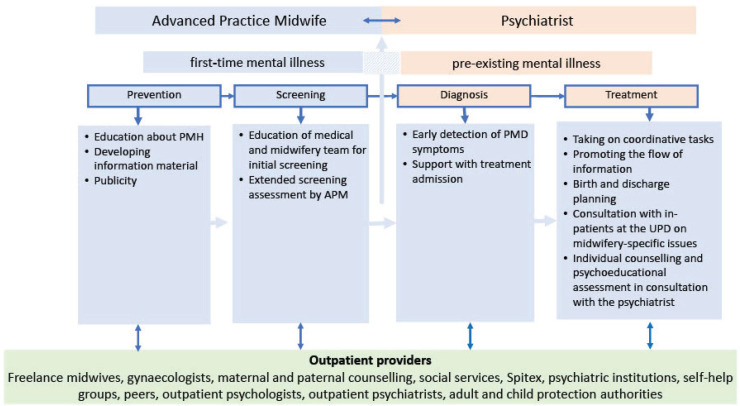
APM model of care

In Step 1 of the original PEPPA framework, the authors used the ‘cancer journey’ model, a flowchart depicting interconnected stages from prevention to treatment, to identify gaps in current care and define interventions. This model was adapted to illustrate the treatment pathway and care for women with PMDs (Figures 1 and 2) and helped to discover the most important gaps and the needs in perinatal mental healthcare, and to determine the scope of the care model. The forthcoming results section will delineate various measures undertaken for Steps 4–7.

After a one-year pilot phase from 1 February 2021 to 31 January 2022, the screening project was evaluated (PEPPA Step 8) in the context of a BFH Master’s thesis. This evaluation study received ethics approval from the Cantonal Ethics Committee for Research, Health and Welfare Directorate of the Canton of Bern, Switzerland, on 27 August 2021 (reference number 2021-00909). The evaluation focused on screening frequencies, results, healthcare professionals’ perceptions of the new APM role, and recommendations for improvement based on practical experience. A multimethod approach, blending qualitative and quantitative designs, assessed the screening project. The steps and related methodological processes are presented in Table 1.

**Table 1:** Methodology for the development of the Advanced Practice Midwife role using the PEPPA framework after a one-year pilot phase from 1 February 2021 to 31 January 2022 in Switzerland

*PEPPA framework steps*	*Aim*	*Method*	*Year*
**Step 1: Define the patient population and the current care model**	Analysis of the current model of care^38^	Qualitative content analysis; interviews with women with pre-existing mental health disorders; data obtained from the MADRE study^17^	2019–2020
**Step 2: Identify stakeholders and recruit participants**	To gain the acceptance and support of key stakeholders, which are critically important to the successful implementation of a new role^38^	Defined stakeholders were persons with a leadership function or with a high level of decision-making authority, which is recommended for successful role development^39,40^	
**Step 3: Determine the need for a new model of care**	To analyze relative strengths and limitations from the perspective of individuals and healthcare providers^38^	Qualitative content analysis; interviews with women with pre-existing mental health disorders and healthcare professionals; data obtained from the MADRE Study^17^	
**Step 4: Identify the priority problems and goals to improve the model of care**	To reach a consensus regarding the most important challenges and problems as well as to identify solutions to improve the care situation^38^	Merging and presenting the results (Steps 1 and 3) to stakeholders (chief doctors with clinic management functions, nursing managers of both hospitals, the head midwife of obstetrics department and a psychiatrist)	
**Step 5: Define the new model of care and the role of APM**	To demonstrate how the goals defined in Step 4 can be adapted for the new care model and role^38^	Strategy meeting with stakeholders (February 2020)	
**Step 6: Plan implementation strategies**	To identify barriers to and facilitators of implementation and define outcomes for the evaluation phase^38^	Engaging stakeholders in planning next steps Development and implementation of the screening project according to best practice recommendations. Collaboration on continuous evaluation planning with Bern University of Applied Sciences (BFH)	2020–2021
**Step 7: Initiate the implementation plan**	Plan the first steps of implementation	With stakeholders, develop a systematic screening pathway and APM guidelines for extended screening assessment Information and education delivered to medical and midwifery teams by the APM	
**Step 8: Evaluate the APM role or new model of care**	Evaluate the screening project	Multimethod approach with qualitative and quantitative phases (Master’s-level midwifery student from BFH)	2022

## RESULTS

### Steps 1 and 3: Qualitative results of the current model of care

Four themes emerged from the qualitative analysis: 1) Experienced inadequate psychiatric treatment in the perinatal period, 2) Community as an important support factor, 3) Experienced challenges, and 4) The need for a care plan provision orientation.

*Experienced inadequate psychiatric treatment in the perinatal period*

All women interviewed reported that they experienced inadequate mental healthcare during the perinatal period. The adapted PMH pathway shows the perceived inadequacies at the various levels of care (Figure 1). Gaps were evident throughout the entire treatment pathway. Healthcare professionals did not verbally provide information about PMH and PMDs, nor were appropriate informational materials made available. None of the interviewees received systematic mental health screenings, even when they reported mental health problems, nor did they receive referrals to psychiatry from an obstetrician or midwife, and the patients had to seek psychiatric help on their own. The women reported that healthcare professionals dismissed mental health changes during pregnancy as a natural occurrence and that they had to consult with different healthcare services until they received a specialized therapy appointment. They experienced long waiting periods for specialized services and were not provided with transitional support while they waited:

*‘… But it was difficult to have the courage, or to realize that you need support, and then you get the information “You can come on Wednesday in three weeks”, and my question was, “Yes, yes, what do I do now? I can’t go work like this, can I? What do I do now?”.’* (WOM1, #00:18:389)

If women were undergoing psychotherapeutic treatment, challenges were identified, including a lack of interprofessional cooperation, insufficient continuity of care, and unclear responsibilities. Health professionals relied on women to manage information exchange, which proved difficult due to the numerous professionals involved.

*Community as an important support factor*

Women and health professionals mentioned the need to establish a support network for mothers experiencing PMDs. Specifically, the importance of the partner’s role in finding and contacting professional help became evident:

*‘I couldn’t manage in this situation to look for something else, somehow or to Google or something. I couldn’t have done that. So, if it hadn’t been for him, I wouldn’t have been able to make this contact, for psychological help.’* (WOM1, #15:19-8)

When resources were lacking, individual situations were more likely to deteriorate, as in the case of a woman who was admitted to a psychiatric hospital and had her child placed in a children’s home.

*Challenges experienced*

In some cases, the challenges experienced by the women took the form of feelings of stigmatization, such as being ‘labeled’ (WOM5, #00:27:05-0) or being treated like a ‘complete idiot’ (WOM4, #00:29:26-1) in the postpartum period. In other cases, they identified feelings of shame and guilt or described how they hid their condition out of fear of losing custody of their children. The fact that the topic was taboo among the mothers themselves was also identified as a challenge. Health professionals identified stigma as a key problem, which often resulted in women seeking help too late, related to the lack of early detection by professionals. The women described the health professionals’ lack of specific expertise as another challenge. They reported that the advice received from the health professionals was not helpful:

*‘And afterwards, when you try to get help from X number of places, you simply get the answer, “Well, you know, this is just a special situation, there’s nothing we can do about it” or simply ZERO understanding, ZERO idea. Afterwards, I had a 30-year-old psychiatrist without children explain to me how it feels to be pregnant.’* (WOM2, #00:10:20-2)

Women also reported that professionals in outpatient services rarely collaborated, reflecting the prevailing fragmented healthcare service. The reasons for the lack of interprofessional collaboration were economic (e.g. roundtable meetings were not billable), a lack of time and resources, a lack of knowledge about other services, and a lack of personal contact with other providers.

*Need for a care plan strategy*

It became evident that a care plan was required during the perinatal period. The interviewed women and healthcare providers expressed parallel concerns about optimized care (Table 2).

**Table 2:** Need for a care plan strategy according to formerly affected women and heath professionals within the Obstetrics Research Department of the Bern University of Applied Sciences Health from 1 February 2021 to 31 January 2022

*Statements by formerly affected women (user perspective)*	*Statements of health professionals (service provider perspective)*
Knowledge transfer about PMD at the beginning of pregnancy	Implementation of systematic PMD screening during pregnancy
Rapid support through a specialized treatment for the perinatal period	Fast, low-threshold access for referring affected women
Desire for a holistic approach and seamless information flow between care providers	Early collaboration with involved health professionals
Continuity of care and a psychiatric reference	Importance of clarifying responsibilities and case guidance
Early postpartum management planning	Need for case management

### Step 4: Identify the priority problems and goals to improve the model of care

In February 2020, a strategy meeting convened with both hospitals’ defined stakeholders (Step 2) to discern priority tasks and goals. The results outlined above (Steps 1 and 3) were presented to all stakeholders and acknowledged as a comprehensive, thematically complete foundation. These findings served as a basis for discussing the prioritized goals and aspects of improved care provision (Step 4). Stakeholders reached a consensus that early PMD detection should be enhanced, PMH should be strengthened, interprofessional collaboration should be promoted, and the coordination of the care process should be optimized. Based on these priorities, the study advanced to Step 5.

### Step 5: Defining the new model of care and the APM role

Given the distinctive position of the APM service at the intersection of psychiatry and obstetrics, it was crucial to clarify and define competencies. Following discussions with stakeholders, the decision was made that the APM service should focus on the dual aspects of health promotion and prevention. Consequently, the core competencies of the APM were established accordingly. For women with PMDs, the role of an APM is to perform tasks delegated by the psychiatrist and assist with coordinating the treatment process (Figure 2). APMs are not authorized to make any psychiatric diagnoses or offer any psychotherapeutic services. They must have at least a Master’s degree and in-depth expertise in the PMH and PMDs to be considered qualified. Hierarchically, APMs work under the leadership of a psychiatrist. In alignment with the goals defined in Step 4, the following core competencies are recommended as part of the APM’s role definition: 1) Education and prevention, 2) Consultation with affected women and families, 3) Referral to other health professionals, and 4) Coordination of treatment for women with a PMD. Figure 2 illustrates the core tasks of APM services in blue, along with collaboration and cooperation with the psychiatrist and outpatient providers (green) throughout the pathway.

### Step 6: Planning implementation

Studies and guidelines recommend systematic PMH screening to ensure timely detection and treatment of patients at risk^1,2^. This was also implied by the women and healthcare providers interviewed (Table 2). Therefore, systematic screening was planned as the initial implementation step. The screening pathway and the extended APM screening assessment were developed with stakeholders and reviewed by the medical directors.

### Step 7: Initiate the implementation plan

The screening comprised two stages: an initial assessment using the Whooley questions (WQs). The WQs are a validated tool to identify the risk for perinatal depression^20^. If either answer was positive, women were asked to complete the Edinburgh Postnatal Depression Scale (EPDS)^21^. A score exceeding ten on the EPDS warranted consultation with a psychiatrist. Due to the availability of APM services, pregnant or postpartum women had the option to undergo an extended screening assessment by the APM if they scored between 10 and 13 points. The results and potential further procedures were discussed with each woman during the APM consultation. Midwives and obstetricians conducted the initial screening during routine antenatal and postpartum consultations. To facilitate this, the APM informed, trained, and supported the medical and midwifery teams.

### Step 8: First evaluation of the APM role and new model of care

During the pilot phase, 509 women were screened, corresponding to 45% of the women who regularly sought antenatal care. Of these women, 13% had positive WQ screenings (Step 1). Subsequently, more than half of the respondents (53%) had positive EPDS results and fulfilled the criteria for extended screening. Of these women, 14% met the criteria (10–12 points) for extended screening assessment by the APM, and 39% met the criteria for direct referral to a psychiatrist (≥12 points). However, most of the women (38%) who required a direct referral to the psychiatrist preferred an initial appointment with the APM. After extended screening with the APM, 35% of the women were referred to the psychiatrist, with an average waiting time of 7.6 days. The evaluation of the EPDS showed that about 13% of the women sometimes had thoughts of harming themselves (EPDS, item 10).

The qualitative evaluation of the pilot study with seven health professionals revealed that cooperation between different specialists, especially the APM, was perceived as helpful and supportive. Especially in difficult situations, staff midwives often turned directly to the APM for support. The obstetrician appreciated the APM’s competence and described a feeling of great relief that allowed him to focus on medical issues during consultations. From the perspective of the professionals, the women and their relatives reported that they viewed addressing mental health issues positively. The professionals reported that due to the routine screening process, their awareness of PMH issues had increased. However, they noted that challenging communication situations with migrant women constituted a barrier to successfully implementing the systematic screening. Additional barriers were limited time resources during counseling and additional documentation efforts related to the evaluation.

## DISCUSSION

This study described the development process and the first steps in implementing and evaluating an APM role and service in the field of PMH. To our knowledge, this is the first specialized midwifery role to be implemented in Switzerland. In contrast to existing APN roles, there is little data on the feasibility, barriers, and facilitators of APM roles in relation to implementation processes^22^. Moreover, although midwives in the UK and Ireland already work as APMs^11^, no studies have specifically described creating an APM role. In Switzerland, the PEPPA framework was successfully used, for example, to develop, implement, and evaluate an APN role in oncology^14^. The clear structure of the PEPPA framework model, along with its sequential steps, proved helpful and goal-oriented for developing an AP role within midwifery.

The role development process revealed gaps along the entire care pathway, including a lack of knowledge among health professionals, poor interdisciplinary communication, highly fragmented PMH care, and a lack of clear responsibilities among healthcare providers. Additionally, the findings revealed long waiting times for women urgently seeking support and a fear of stigmatization on the part of the affected women. A systematic review and meta-synthesis described similar factors of inadequate healthcare provision that impede timely access to services^23^. In Switzerland, these factors are exacerbated by the absence of national programs, unlike in other countries, to support specialized PMH care for women^24^. Consequently, these endeavors in role development have illuminated pertinent shortcomings, underscoring the necessity for revisions to ensure effective and efficient healthcare provision for affected women.

### Responsibility and liability in the APM model

The developed APM service serves as a bridge between obstetrics and psychiatry, and accordingly, it is based on a delegation model. The assigned APM competencies lie within health promotion and prevention (Figure 2). When working with a woman with a PMD diagnosis, the APM works with other involved health professionals to coordinate the woman’s care and treatment (Figure 2) and regularly consults with a psychiatrist. It has been shown that for patients with mental health disorders, collaboration between various health professionals from different disciplines is effective and helpful^25,26^. Further, such collaboration has been recommended specifically for PMH care^27,28^. In this study, cooperation with the University Hospital of Psychiatry and Psychotherapy played an important role in developing the presented APM role, as the hospital’s psychiatrist acted as the APM’s clinical supervisor. With regard to delegation models, trust is identified as one of the key factors in fostering a sustainable working relationship between the APM and various stakeholders^29^. To ensure confidence, involving stakeholders and clarifying and defining competencies was crucial.

After role development, it became evident that immediate implementation of all aspects of the developed model in the clinic would not be feasible. This aligns with the typical clinical implementation processes, as noted by the authors of PEPPA^16^. The full development of a new role within a new model requires flexible oscillation between Steps 6 and 7 to stabilize the structure and implement long-term monitoring of the role and care model^16^. Based on the available personal and structural resources and strong agreement with stakeholders, the first implementation step was the systematic PHM screening. This approach aligns with international recommendations to o prioritize the early detection of PMDs^1,2^. Healthcare professionals have reported that, since the implementation of screening in clinical care, they have become more aware of potential PMDs and feel more secure when systematically assessing PMH. Implementing screening can also enhance awareness of mental health among healthcare professionals and women themselves^30^.

### Success factors for the implementation of new care models

The education and training of professional teams and the provision of necessary resources are crucial for the successful implementation of midwifery projects^31^. Therefore, a manual was provided to ensure standardized screening procedures, and all staff were informed and trained about the process by the APM. Despite the successful introduction of the APM, the first screening with the WQs was only performed in 45% of women, possibly due to incomplete integration into daily routines. A similar phenomenon was observed in another study, where the number of screenings started to increase in the second year after implementation^32^. Therefore, continuous evaluation of the screening is recommended. According to a recent study, the screening program described here meets the current recommendations^33^. In addition to the resources required for screening, these recommendations include timely diagnosis, appropriate treatment, and comprehensible action paths within the health organization. In the long-term, systematic screening for PMDs is expected to lead to improved health outcomes for women and their children^33,34^. It can potentially reduce exceedingly high long-term costs related to unidentified PMDs^35^. Recently published data about the causes of maternal mortality in the perinatal period from the USA and UK highlight the negative consequences of untreated maternal mental health conditions, which are considered the main causes of maternal deaths during this period in both countries^36,37^. Although motherhood is not generally associated with suicide in society, 13% of the women in our sample reported suicidal thoughts. This finding underlines the importance of screening as an early detection method followed by early treatment.

### Strengths and limitations

A major strength of this study is its systematic role development based on data from the MADRE project. Furthermore, this is the first time the PEPPA framework model has been used in the midwifery sector in APM development, implementation, and evaluation. Additionally, continued cooperation with the University of Applied Sciences ensures the further development and evaluation of the program. The study’s limitations include the small amount of data collected during role development and the specificity of the data to a particular location within a university hospital. Nevertheless, this study is a model for others seeking to develop APM services in their contexts.

## CONCLUSIONS

This study presented the innovative process of developing and implementing an APM model in the field of PMH using the PEPPA framework. Although the APM role is not legally regulated in Switzerland, and there is no national consensus on the required competencies for APMs with a Master’s degree, the role was successfully implemented due to medical and institutional support and the determination of the stakeholders. The introduction of APM services increased awareness among hospital staff about the mental health issues of pregnant women, a systematic PMH screening was introduced, and the identification of high-risk pregnancies increased. Further measures from the APM model are being introduced along the care pathway to improve care for affected women and families. This project continues to be evaluated to gain new insights into the role of APMs. This study’s findings are intended to assist midwives with Master’s degrees in establishing corresponding services using the PEPPA framework model and thus effectively contribute to the health of mothers and children.

## Data Availability

The data supporting this research are available from the authors on reasonable request.

## References

[1] World Health Organization. Guide for integration of perinatal mental health in maternal and child health services. WHO; 2022. Accessed June 9, 2024. https://iris.who.int/bitstream/handle/10665/362880/9789240057142-eng.pdf?sequence=1

[2] Howard LM, Khalifeh H. Perinatal mental health: a review of progress and challenges. World Psychiatry. 2020;19(3):313-327. doi:10.1002/wps.20769 32931106 PMC7491613

[3] Berger A, Bachmann N, Signorell A, et al. Perinatal mental disorders in Switzerland: prevalence estimates and use of mental-health services. Swiss Med Wkly. 2017;147:w14417. doi:10.4414/smw.2017.14417 28322424

[4] Gentile S. Untreated depression during pregnancy: Short- and long-term effects in offspring. A systematic review. Neuroscience. 2017;342:154-166. doi:10.1016/j.neuroscience.2015.09.001 26343292

[5] Nakic Radoš S, Ayers S, Horsch A. Editorial: From childbearing to childrearing: Parental mental health and infant development. Front Psychol. 2022;13:1123241. doi:10.3389/fpsyg.2022.1123241 36687935 PMC9850224

[6] Chew-Graham C, Chamberlain E, Turner K, Folkes L, Caulfield L, Sharp D. GPs’ and health visitors’ views on the diagnosis and management of postnatal depression: a qualitative study. Br J Gen Pract. 2008;58(548):169-176. doi:10.3399/bjgp08x277212 18399021 PMC2249792

[7] Lommerse KM, Mérelle S, Rietveld AL, Berkelmans G, van den Akker T; Netherlands Audit Committee Maternal Mortality and Morbidity. The contribution of suicide to maternal mortality: A nationwide population-based cohort study. BJOG. 2024. doi:10.1111/1471-0528.17784 38344899

[8] Clement S, Schauman O, Graham T, et al. What is the impact of mental health-related stigma on help-seeking? A systematic review of quantitative and qualitative studies. Psychol Med. 2015;45(1):11-27. doi:10.1017/s0033291714000129 24569086

[9] Patel V, Chisholm D, Parikh R, et al. Addressing the burden of mental, neurological, and substance use disorders: key messages from Disease Control Priorities, 3rd edition. Lancet. 2016;387(10028):1672-1685. doi:10.1016/s0140-6736(15)00390-6 26454360

[10] Schmied V, Johnson M, Naidoo N, et al. Maternal mental health in Australia and New Zealand: a review of longitudinal studies. Women Birth. 2013;26(3):167-178. doi:10.1016/j.wombi.2013.02.006 23583667

[11] Begley C, Murphy K, Higgins A, et al. Evaluation of Clinical Nurse and Midwife Specialist and Advanced Nurse and Midwife Practitioner Roles in Ireland (SCAPE): Final Report. National Council for the Professional Development of Nursing and Midwifery; 2010. Accessed June 9, 2024. https://www.tara.tcd.ie/bitstream/ handle/2262/68341/SCAPE_Final_Report_13th_May.pdf?sequence=1&isAllowed=y

[12] Htay M, Whitehead D. The effectiveness of the role of advanced nurse practitioners compared to physician-led or usual care: A systematic review. Int J Nurs Stud Adv. 2021;3:100034. doi:10.1016/j.ijnsa.2021.100034 38746729 PMC11080477

[13] Cignacco E, Schlenker A, Ammann-Fiechter S, et al. Advanced Midwifery Practice in Switzerland: Development and challenges. Eur J Midwifery. 2024;8. doi:10.18332/ejm/185648 PMC11034162 38650967

[14] Serena A, Castellani P, Fucina N, et al. The role of advanced nursing in lung cancer: A framework based development. Eur J Oncol Nurs. 2015;19(6):740-746. doi:10.1016/j.ejon.2015.05.009 26059323

[15] Boyko JA, Carter N, Bryant-Lukosius D. Assessing the spread and uptake of a framework for introducing and evaluating advanced practice nursing roles. Worldviews Evid Based Nurs. 2016;13(4):277-284. doi:10.1111/wvn.12160 27074416

[16] Bryant-Lukosius D, Dicenso A, Browne G, Pinelli J. Advanced practice nursing roles: development, implementation and evaluation. J Adv Nurs. 2004;48(5):519-529. doi:10.1111/j.1365-2648.2004.03234.x 15533090

[17] Berger A, Schenk K, Ging A, Walther S, Cignacco E. Perinatal mental health care from the user and provider perspective: protocol for a qualitative study in Switzerland. Reprod Health. 2020;17(1):26. doi:10.1186/s12978-020-0882-7 32066475 PMC7027089

[18] Handbuch für f4transkript und f5transkript (Version 7 - April 2018). audiotranskription. Accessed June 9, 2024. https://www.audiotranskription.de/manuals/f4transkript_v7_de.pdf

[19] Braun V, Clarke V. Using thematic analysis in psychology. Qualitative Research in Psychology. 2006;3(2):77-101. doi:10.1191/1478088706qp063oa

[20] Howard LM, Ryan EG, Trevillion K, et al. Accuracy of the Whooley questions and the Edinburgh Postnatal Depression Scale in identifying depression and other mental disorders in early pregnancy. Br J Psychiatry. 2018;212(1):50-56. doi:10.1192/bjp.2017.9 29433610 PMC6457164

[21] Cox JL, Holden JM, Sagovsky R. Detection of postnatal depression: development of the 10-item Edinburgh Postnatal Depression Scale. Br J Psychiatry. 1987;150:782-786. doi:10.1192/bjp.150.6.782 3651732

[22] Goemaes R, Shawe J, Beeckman D, Decoene E, Verhaeghe S, Van Hecke A. Factors influencing the implementation of advanced midwife practitioners in healthcare settings: A qualitative study. Midwifery. 2018;66:88-96. doi:10.1016/j.midw.2018.08.002 30165272

[23] Sambrook Smith M, Lawrence V, Sadler E, Easter A. Barriers to accessing mental health services for women with perinatal mental illness: systematic review and meta-synthesis of qualitative studies in the UK. BMJ Open. 2019;9(1):e024803. doi:10.1136/bmjopen-2018-024803 PMC6347898 30679296

[24] Amiel Castro RT, Schroeder K, Pinard C, et al. Perinatal mental health service provision in Switzerland and in the UK. Swiss Med Wkly. 2015;145:w14011. doi:10.4414/smw.2015.14011 25701656

[25] Gilbody S, Bower P, Fletcher J, Richards D, Sutton AJ. Collaborative care for depression: a cumulative meta-analysis and review of longer-term outcomes. Arch Intern Med. 2006;166(21):2314-2321. doi:10.1001/archinte.166.21.2314 17130383

[26] Archer J, Bower P, Gilbody S, et al. Collaborative care for depression and anxiety problems. Cochrane Database Syst Rev. 2012;10:CD006525. doi:10.1002/14651858.CD006525.pub2 23076925 PMC11627142

[27] National Institute for Health and Care Excellence. Antenatal and postnatal mental health: clinical management and service guidance. National Institute for Health and Care Excellence. December 17, 2014. Updated February 11, 2020. Accessed June 9, 2024. https://www.nice.org.uk/guidance/cg192/resources/antenatal-and-postnatal-mental-health-clinical-management-and-service-guidance-pdf-35109869806789 31990493

[28] Centre of Perinatal Excellence. Mental health care in the perinatal period: Australian Clinical Practice Guideline. Centre of Perinatal Excellence; 2017. Accessed June 9, 2024. https://cope.org.au/wp-content/uploads/2017/10/Final-COPE-Perinatal-Mental-Health-Guideline.pdf

[29] Lauber E, Kindlimann A, Nicca D, et al. Integration of an advanced practice nurse into a primary care practice: a qualitative analysis of experiences with changes in general practitioner professional roles in a Swiss multiprofessional primary care practice. Swiss Med Wkly. 2022;152:w30199. doi:10.4414/smw.2022.w30199 35816630

[30] Vik K, Aass IM, Willumsen AB, Hafting M. Experiences with the routine use of the Edinburgh Postnatal Depression Scale from health visitors’ and midwives’ perspectives - An exploratory qualitative study. Midwifery. 2021;100:103017. doi:10.1016/j.midw.2021.103017 33971380

[31] Loytved CAL, Hasenberg G, Brendel K, et al. Implementation in nursing and midwifery. A scoping review / Implementationsprojekte in der Pflege und Hebammenarbeit. Scoping review. International Journal of Health Professions. 2017;4(2):122-136. doi:10.1515/ijhp-2017-0021

[32] Miller ES, Jensen R, Hoffman MC, et al. Implementation of perinatal collaborative care: a health services approach to perinatal depression care. Prim Health Care Res Dev. 2020;21:e30. doi:10.1017/s1463423620000110 32907689 PMC7503171

[33] Waqas A, Koukab A, Meraj H, et al. Screening programs for common maternal mental health disorders among perinatal women: report of the systematic review of evidence. BMC Psychiatry. 2022;22(1):54. doi:10.1186/s12888-022-03694-9 PMC8787899 35073867

[34] Howard LM, Abel KM, Atmore KH, et al. Perinatal mental health services in pregnancy and the year after birth: the ESMI research programme including RCT. Programme grants for applied research. 2022;10(5). doi:10.3310/CCHT9881 35700306

[35] Bauer A, Parsonage M, Knapp M, Iemmi V, Adelaja B. The costs of perinatal mental health problems. Centre for Mental Health and London School of Economics; 2014. Accessed June 9, 2024. https://www.researchgate.net/publication/267924828_The_costs_of_perinatal_mental_health_problems

[36] Knight M, Bunch K, Patel R, et al. Saving lives, improving mothers’ care core report–lessons learned to inform maternity care from the UK and Ireland Confidential Enquiries into Maternal Deaths and Morbidity 2018–20. Oxford: National Perinatal Epidemiology Unit, University of Oxford; 2022. Accessed June 9, 2024. https://allcatsrgrey.org.uk/wp/download/obstetrics/MBRRACE-UK_Maternal_MAIN_Report_2022_v10.pdf

[37] Trost S, Beauregard J, Chandra G, et al. Pregnancy-Related Deaths: Data from Maternal Mortality Review Committees in 36 States, 2017-2019. Centers for Disease Control and Prevention, US Department of Health and Human Services; 2022. Accessed June 9, 2024. https://www.cdc.gov/maternal-mortality/media/pdfs/Pregnancy-Related-Deaths-Data-MMRCs-2017-2019-H.pdf

[38] Bryant-Lukosius D, Dicenso A. A framework for the introduction and evaluation of advanced practice nursing roles. J Adv Nurs. 2004;48(5):530-540. doi:10.1111/j.1365-2648.2004.03235.x 15533091

[39] DiCenso A, Bryant-Lukosius D, Martin-Misener R, et al. Factors enabling advanced practice nursing role integration in Canada. Nurs Leadersh. 2010;23:211-238. doi:10.12927/cjnl.2010.22279 21478695

[40] Wisur-Hokkanen C, Glasberg AL, Mäkelä C, Fagerström L. Experiences of working as an advanced practice nurse in Finland - the substance of advanced nursing practice and promoting and inhibiting factors. Scand J Caring Sci. 2015;29(4):793-802. doi:10.1111/scs.12211 25656095

